# The Instrumented Stand and Walk (ISAW) test to predict falls in older men

**DOI:** 10.1007/s11357-022-00675-4

**Published:** 2022-10-27

**Authors:** Fay B. Horak, Amy Laird, Patricia Carlson-Kuhta, Melanie Abrahamson, Martina Mancini, Eric S. Orwoll, Jodi A. Lapidus, Vrutangkumar V. Shah

**Affiliations:** 1 Department of Neurology, Oregon Health & Science University, 3181 SW Sam Jackson Park Rd, OP-32, Portland, OR 97239 USA; 2 APDM Wearable Technologies, Clario Company, 2828 S Corbett Ave, #135, Portland, OR 97201 USA; 3 School of Public Health, OR Health & Science University-Portland State University, 3181 SW Sam Jackson Park Rd, Portland, OR 97239 USA; 4 Department of Endocrinology, Oregon Health & Science University, 3181 SW Sam Jackson Park Rd, Portland, OR 97239 USA

**Keywords:** Prospective falls, Fall risk assessment, Balance, Gait, Wearable sensors

## Abstract

Objective measures of balance and gait have the potential to improve prediction of future fallers because balance and gait impairments are common precursors. We used the Instrumented Stand and Walk Test (ISAW) with wearable, inertial sensors to maximize the domains of balance and gait evaluated in a short test. We hypothesized that ISAW objective measures across a variety of gait and balance domains would improve fall prediction beyond history of falls and better than gait speed or dual-task cost on gait-speed. We recruited 214 high-functioning older men (mean 82 years), of whom 91 participants (42.5%) had one or more falls in the 12 months following the ISAW test. The ISAW test involved 30 s of stance followed by a 7-m walk, turn, and return. We examined regression models for falling using 17 ISAW metrics, with and without age and fall history, and characterize top-performing models by AUC and metrics included. The ISAW test improved distinguishing between future fallers and non-fallers compared to age and history of falls, alone (AUC improved from 0.69 to 0.75). Models with 1 ISAW metric usually included a postural sway measure, models with 2 ISAW measures included a turning measure, models with 3 ISAW measures included a gait variability measure, and models with 4 or 5 measures added a gait initiation measure. Gait speed and dual-task cost did not distinguish between fallers and non-fallers in this high-functioning cohort. The best fall-prediction models support the notion that older people may fall due to a variety of balance and gait impairments.

## Introduction

The persistence of high rates of falling in older adults, despite clinical fall-risk assessments [1], suggests that more specific measures of fall risk are needed. Fall history is the strongest predictor of future falls [2]. However, information about number and severity of falls over a previous year from older patients’ memories can be inaccurate, especially for patients with memory problems and those who fall very often [3]. Also, people who have not yet fallen may fall in the next year [3, 4]. Slow gait speed has also been associated with falls in older people although robust, high-functioning older adults with normal gait speed also fall. Thus, quantification of subtle, specific balance and gait characteristics with a quick and objective test may be a useful addition to fall history and gait speed to identify fall risk in high-functioning older adults.

The most common reasons for falls (and modifiable risk factors) are balance and gait impairments [2]. Balance and gait have several different domains affected by aging and, therefore, likely to contribute in different ways to fall risk [5]. Each balance domain, such as anticipatory postural adjustments (APAs; postural adjustments that precede voluntary movements, such as step initiation, to maintain postural stability), postural sway in stance, and dynamic balance (lateral trunk control) while turning are thought to be nonredundant and represent different neural control circuits and different potential reasons for falls [6]. In addition, postural sway metrics have been shown to better predict fall risk than stopwatch measures, such as standing on one foot [7]. Like balance, gait has many independent, measurable domains such as gait variability (e.g. stride-time variability) and spatial (e.g. stride length), temporal (e.g. double-support time), and upper body (e.g. trunk range of motion) domains [8]. Many instrumented measures of gait, such as double-support time and stride-time variability, may predict future fallers better than gait speed [9]. In addition, enhancement of gait impairments during an attention-demanding, cognitive dual-task may be even more sensitive to fall risk than single-task gait [10]. Rehabilitation of balance and gait is recommended to prevent falls, but effective rehabilitation requires assessment of the specific domain of balance and gait to target [11].

To capture multiple domains of balance and gait in one test, we used an Instrumented Stand and Walk Test (ISAW), a short balance and gait task, using wearable inertial sensors [12]. The test results in metrics from several different domains of mobility: postural sway in quiet stance, anticipatory postural adjustments associated with step initiation, quality of turning 180°, and 4 domains of gait characteristics that have been shown to be independent factors (spatial, temporal, variability, and upper body) [6, 8]. Identifying the set of balance and gait domains that are best related to falls will help to (1) better understand underlying mechanisms for falls, (2) design a concise test protocol, and (3) focus rehabilitation on specific balance and gait domains.

Our goal was to identify which combinations of ISAW metrics, reflecting different domains of gait and balance, would best help separate prospective fallers from non-fallers [6, 13–15]. We hypothesized that composite models of objective balance/gait metrics across different domains in the ISAW test would predict who would fall in the next 12 months. These models tell us which specific domains of gait and balance best predict falls in high-functioning older adults. We also hypothesized that using metrics from the ISAW test would improve the ability to predict who would fall in the following year, beyond clinical predictors such as history of falls and age. And additionally predict better than gait speed or dual-task cost on gait speed in our high-functioning cohort of men in their 9th decade of life.

## Materials and methods

### Participants

**Fig. 1:**
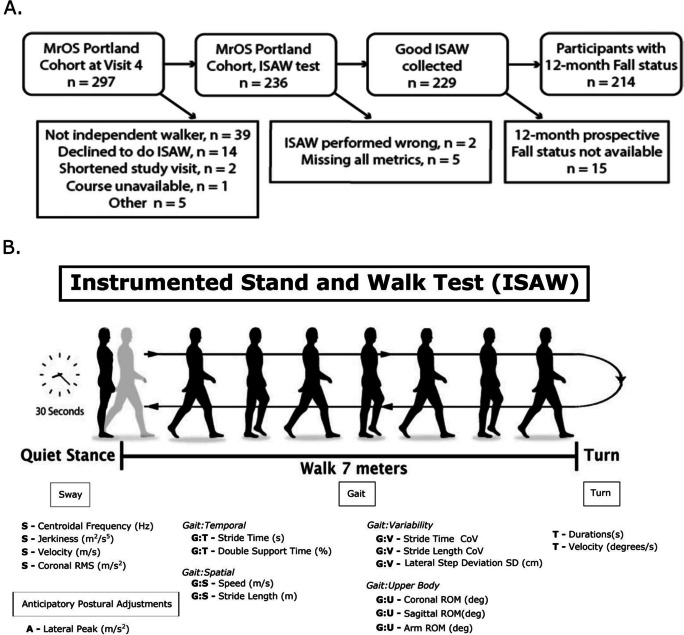
**A** Flowchart of number of participants in the study and reasons for dropouts. **B** Summary of ISAW protocol, mobility domains, and metrics collected. Participants stood for 30 s, walked 7 m, turned around after crossing a line on the floor and returned 7 m to the starting location. The balance and gait metrics used are listed at the bottom, and belong to 4 primary mobility domains: postural Sway during quiet stance, gait over 7 m × 2, turning 180°, and anticipatory postural adjustments (APAs) prior to step initiation. Gait metrics were further divided into independent factors of temporal, spatial, variability, and upper body metrics. A anticipatory, S sway, G:T gait/temporal, G:S gait/speed, G:V gait/variability, G:U gait/upper body, T turn

We recruited community-dwelling, older participants from one site of the largest longitudinal study of falls in older men, The Osteoporotic Fractures in Men (MrOS; https://mrosonline.ucsf.edu) [16, 17]. MrOS has 20 years of data on falls and fall injury, physical performance, health status, as well as falls, in men who are now in their 80 s. When originally recruited between 2000 and 2002, the men in MrOS were 65 years of age or older, able to walk without assistance, and without neurological disorders or joint replacements. The Portland-site MrOS cohort for visit 4 was recruited between May 2014 and May 2016 and was able to stand and walk for 2 min without the use of a cane or other assistive device, although a few used a cane occasionally in daily life. We tested 214 participants, 11 of whom were unable to travel to the site so they were given the ISAW test in their homes. See Fig. 1A for details of participant numbers. The methods were performed in accordance with relevant guidelines and regulations and approved by the Oregon Health & Science University, Institutional Review Board. All participants were given a written consent form to read, had the study verbally explained to them, and, after having any questions answered, documented their consent by signing the informed consent form.

### ISAW test

Participants wore 6 inertial measurement units (Opals by APDM Wearable Technologies, a Clario company) (on each foot, the low back, sternum and both wrists), and data were collected using the Mobility Lab system. The ISAW test consists of (1) standing for 30 s with eyes open, hands at sides with stance width standardized using a foot template [18], (2) step initiation, (3) walking 7 m, (4) turning 180° over a line on the ground, and (5) walking 7 m back to the start line (Fig. 1B [12]). This test was repeated with a concurrent, cognitive dual task, in which the participant was asked to count backward by 3 s (starting from a standard, 3-digit number). Subjects first practiced counting backward by 3 s from a different 3-digit number while sitting for 1 min.

We focused on the following metrics from 7 different domains of mobility: (1) sway during standing balance (e.g. area, velocity, frequency), (2) anticipatory postural adjustments for step initiation (peak lateral trunk range), (3) gait spatial measures (e.g. stride length), (4) gait temporal measures (e.g. cadence, double-support time), (5) upper body gait (e.g. trunk range), (6) gait variability (e.g. stride time variability), and (7) turning quality (e.g. peak turn velocity, number of steps). We a priori considered 17 ISAW measures out of over 100 ISAW potential measures for this study by selecting valid and reliable, nonredundant (not highly correlated) measures from each domain [13–15, 19] (Fig. 1B). We used the commercial gait analysis algorithms included in Mobility Lab™, Version 2 (APDM Wearable Technologies-a Clatrio company, Inc., Portland, Oregon) [12] to extract 17 ISAW measures which have been validated previously [14, 20–22].

### Clinical measures

Demographic and clinical characteristics recorded at MrOS Visit 4 included age, height, weight, marital status, living arrangement, self-rated quality of health, and use of walking aids. The Physical Activity Scale for the Elderly (PASE), a patient-reported scale of physical activity over the past month, was administered. The PASE has a maximum total score (highest physical activity) of 793 and an average score of 101 for men > 70 years [23]. Other clinical tests included (1) walking speed based on two stopwatch trials of a 6-m, natural-pace walk, (2) ability to complete 5 chair stands, and (3) bilateral grip strength.

### Falls

To define a prospective faller, we utilized the MrOS postcards that were sent out every 4 months to inquire if any falls had occurred. We defined as a participant with at least one reported fall in the 12-month period following the ISAW test at MrOS visit 4. Missing data on falls (such as a postcard not being returned) were handled as follows: participants for whom fall status in the 12-month period following the ISAW test could be determined were included in the analysis, and others were not included.

Cognitive function of both fallers and non-fallers was in the normal range for their age. See Teng Modified Mini-Mental State (3MS) in Table 1. Numbers of reported falls in each 4-month interval were used to determine fall status over the 12-month period after the ISAW test.

**Table 1:** Demographic and clinical characteristics at MrOS Visit 4 separated by fall status in the 12-month period following ISAW test. Results given as median (interquartile range), or percent (frequency), as appropriate

Characteristic	All with known fall status (*n* = 214)	Non-fallers (*n* = 123)	Fallers (*n* = 91)
Age, years	83 (80, 87)	83 (80, 86)	84 (80, 89)
BMI, kg/m^2^	25.8 (23.8, 28.3)	25.5 (23.5, 28.4)	26.0 (24.4, 28.0)
Living situation:			
Alone in community	22.4 (48)	20.3 (25)	25.3 (23)
With others in community	73.8 (158)	74.8 (92)	72.5 (66)
Assisted living	3.7 (8)	4.9 (6)	2.2 (2)
Race/ethnicity			
White	91.6 (196)	91.1 (112)	92.3 (84)
African-American	3.3 (7)	4.1 (5)	2.2 (2)
Asian	2.8 (6)	2.4 (3)	3.3 (3)
Hispanic	0.5 (1)	0.8 (1)	0.0 (0)
Other	1.9 (4)	1.6 (2)	2.2 (2)
Self-rated quality of health			
Excellent	43.5 (93)	44.7 (55)	41.8 (38)
Good	48.1 (103)	46.3 (57)	50.5 (46)
Fair, poor, or very poor	8.4 (18)	8.9 (11)	7.7 (7)
Teng Modified Mini-Mental State (3MS)	96.5 (92, 99)	97 (92, 99)	96 (92, 99)
No walking aids	95.7 (202)	96.7 (117)	94.4 (85)
PASE score	127 (80, 171)	132 (89, 175)	111 (67, 169)
6-m walking speed, m/s	1.19 (1.04, 1.33)	1.22 (1.08, 1.34)	1.15 (1.01, 1.29)
% able to complete 5 chair stands	98.5 (193)	99.1 (113)	97.6 (80)
Grip strength, kg (avg of left and right)	33.5 (29.0, 39.0)	36.0 (30.8, 39.3)	32.5 (27.0, 36.0)
Falls in 12 months before ISAW test:			
% who fell once or more	37.4	26.0	52.7
Number of falls (total)*	165	40	125
Falls in 12 months after ISAW test:			
% who fell once or more	42.5	0.0	100.0
Number of falls (total)*	227	0	227

^*^Details about fall numbers are in Fig. 2

### Statistical analysis

Given our hypothesis that ISAW metrics across different mobility domains could help predict falls and the limitation of our sample size, we chose a modeling approach that averaged across many statistical models, rather than choosing the “best” model. We ran a set of logistic regression models on prospective fall status (fallers versus non-fallers) in the 12 months following ISAW: covariates included age at MrOS visit 4, estimated number of falls in the year before ISAW (continuous value), and all possible subsets of the top-performing balance and gait metrics (log-transforming skewed metrics). Since there were 17 ISAW metrics under consideration, we had 2^17^ = 131,072 potential models. We computed the Bayesian Information Criterion (BIC) [24], a measure of model fit, and assigned a “weight” to the model based on the value of the BIC [25, 26]. We combined coefficient estimates for the ISAW metrics from each model, using the weights on the models, to yield Bayesian model-averaged, coefficient estimates, referred to as the BIC-weighted model average. Additionally, for each ISAW metric, we computed a Bayesian posterior probability of inclusion in the “true” model. These posterior probabilities can be interpreted as measures of importance of each ISAW metric in discriminating fallers from non-fallers, with and without including age and fall history in the model. We also compared the 17 ISAW metrics between non-fallers and fallers using Student *T* tests.

We examined how well ISAW metrics could aid in distinguishing fallers from non-fallers with and without information on fall history and age. Comparing each analysis across these two scenarios: (a) ISAW metrics alone and (b) ISAW metrics + fall history + age, allowed us to see which ISAW metrics and domains were amplified in their importance when fall history and age were included in the model. In order to see the impact of including ISAW metrics, we compared ROC curves from the model that included fall history and age only, the BIC-weighted average of all possible models, and the top-performing models with 3 ISAW metrics plus fall history and age. To see the impact of including dual-task cost on gait speed and turning speed in the model, we added dual-task cost calculated as: dual-task cost [%] = 100 × (dual-task metric − single-task metric) / single-task metric. All analyses were done in Stata/IC 15.1 for Windows.

### Data availability

Information on accessing the data is available from https://mrosonline.ucsf.edu/

## Results

### Participant characteristics

In total, 236 participants from the Portland MrOS site participated in this ancillary ISAW study and 229 contributed usable ISAW data. For 15 of these 229 participants, fall status in the 12 months following the ISAW test was indeterminate due to missing data (Fig. 1A). Reasons for missing information on falls included death, missing postcards, or postcards with missing information. Hence, 214 participants were included in our fall prediction analysis. The health and demographic information were collected at the MrOS Study Visit 4. The ISAW test was also administered at visit 4 or within the following month, in 206 of the 214 (96.3%).

Table 1 summarizes characteristics of the 214 high functioning older men in whom we predicted falls in the following 12 months. Most participants lived in the community (96.3%), either alone or with a family member. Only 8 participants (3.7%) were living in an assisted living facility. The majority (91.6%) reported their quality of health at visit 4 as either “excellent” or “good” and most (94.4%) did not use walking aids. Only a few participants reported comorbidities that may contribute to fall risk, such as Parkinson’s disease (*n* = 5, all prospective fallers), stroke (*n* = 22, 8/22 = 36% prospective fallers), or dementia (*n* = 10, 6/10 = 60% prospective fallers). Median PASE score for self-reported activity level was 127 out of a maximum of 793 (interquartile range 80–171, a high level of reported activity for their age) [23].

Differences between non-fallers and fallers across the 17 ISAW metrics are summarized in Table 2. Fallers showed slower sway velocity, slower gait speed, shorter strides, more variable stride time, and slower turning velocity.

**Table 2:** Mean and SD *for* each of the ISAW measures with the p-value between fallers and non-fallers

ISAW metric	Non-fallers (*n* = 123)		Fallers (*n* = 91)		*p*-value
	Mean	SD	Mean	SD	
*S* — centroidal frequency (Hz)*	− 0.068	0.259	− 0.125	0.239	0.104
*S* — jerkiness (m^2^/s^5^)*	0.361	1.032	0.367	0.979	0.965
*S* — velocity (m/s)*	− 2.085	0.595	− 1.888	0.566	**0.015**
*S* — coronal RMS (m/s^2^)*	− 3.649	0.602	− 3.540	0.510	0.164
*A* — lateral peak (m/s^2^)	0.381	0.164	0.422	0.180	0.107
*G:T* — stride time (s)	1.119	0.092	1.138	0.095	0.150
*G:T* — double support time (%)	22.21	3.519	23.09	3.736	0.079
*G:S* — speed (m/s)	1.064	0.176	1.000	0.187	**0.011**
*G:S* — stride length (m)	1.175	0.152	1.125	0.187	**0.033**
*G:V* — stride time CoV	0.031	0.013	0.035	0.015	**0.045**
*G:V* — stride length CoV	0.084	0.062	0.091	0.055	0.383
*G:V* — lateral step deviation SD (cm)	4.079	1.194	4.333	1.461	0.164
*G:U* — coronal ROM (deg)	5.436	2.128	5.330	2.115	0.719
*G:U* — sagittal ROM(deg)	4.307	1.258	4.200	1.183	0.528
*G:U* — arm ROM (deg)	40.66	14.62	38.35	13.76	0.242
*T* — durations (s)	2.354	0.491	2.389	0.471	0.601
*T* — velocity (deg/s)	169.4	46.00	154.80	38.90	**0.016**

*P* < 0.05 is in bold

*S* sway, *A* anticipatory postural adjustment, *G:T* gait/temporal, *G:S* gait/spatial (pace), *G:V* gait/variability, *G:U* gait/upper body, *T* turning

^*^log-transformed metric (base e)

### Falls

**Fig. 2:**
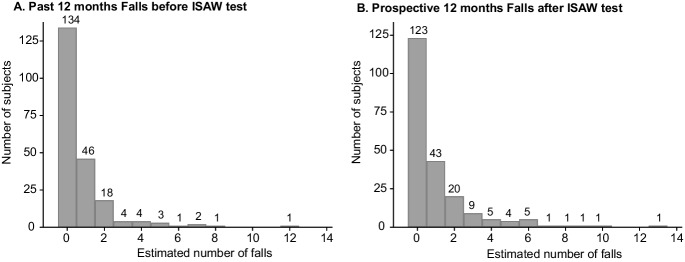
**A** Number of falls participants had in the 12 months prior to the ISAW test among the 214 participants. **B** Number of falls participants had in the 12 months following ISAW test among the 214 participants

During the 12 months before the ISAW test, 80 of the 214 participants (37.4%) reported at least one fall, and the total number of reported falls in this period was 165. The maximum number of reported falls per participant in the year prior to the ISAW test was 12 (Fig. 2A). The total number of prospective falls over 12 months in our cohort was 227, and the maximum number of reported falls per participant was 13 (frequency distribution in Fig. 2B). Ninety-one out of the 214 participants included in our analysis (42.5%) had one or more estimated falls in the 12 months following the ISAW test. Fall status in the 12 months before and after the ISAW test was able to be determined for all 214 participants. Unsurprisingly, fall history was associated with future falls: 67.9% of participants who did not fall in the 12 months prior to the ISAW also did not fall in the following 12 months, while 60.0% of those who fell in the 12 months prior to the ISAW also fell in the following 12 months.

### Prediction of falls

**Fig. 3:**
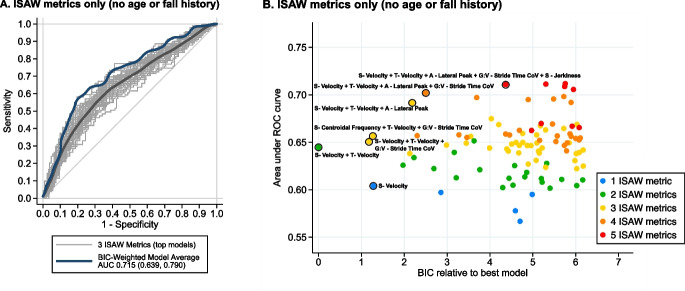
ROC curves for discriminating between fallers and non-fallers. A. ISAW metrics only (no fall history and age). Best-fitting models that include 3 ISAW metrics are shown in light grey, with smoothed average shown overlaid in thick dark grey. Smoothed curve for BIC-weighted model average is shown in thick dark blue. B. Plot of best-fitting 100 models for distinguishing future fallers from non-fallers based on AUC versus best-fitting models (Bayesian Information Criterion (BIC); best is zero on this scale) for ISAW metrics alone (color-coded by number of ISAW metrics. The strongest models are circled in black with a list of the included ISAW metrics. Notice that sway (S) was the most commonly included ISAW domain

ISAW metrics, alone, were able to distinguish future fallers from non-fallers quite well with the area under the curve (AUC) of the receiver operating characteristic (ROC) curve = 0.715 (95% confidence interval (0.639, 0.790)) for the BIC-weighted model average), even without history of falls or age (Fig. 3A). When three ISAW metrics were included in the model, the median AUC among 3-metric models in the top 100 was 0.647, and the maximum AUC over all 3-metric models was 0.692. Figure 3B shows the tradeoff between model performance as measured by AUC and model fit as measured by the Bayesian Information Criterion (BIC), as well as the impact of including additional ISAW metrics in the model, for the 100 top-performing models in this scenario. The best-fitting ISAW model had two metrics, and including up to four ISAW metrics conferred gains in AUC without substantial loss of model fit.

**Fig. 4:**
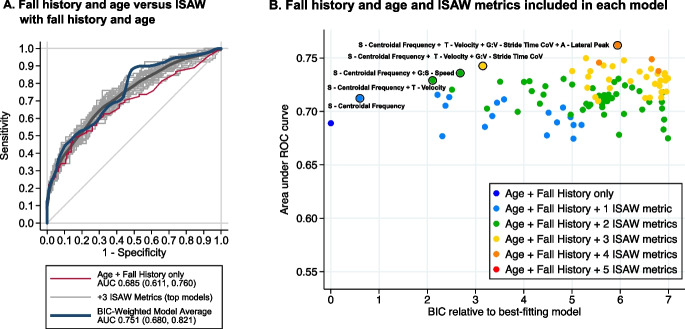
ROC curves for discriminating between fallers and non-fallers with each model including fall history and age with ISAW metrics, and model that includes fall history and age, only, is shown in red (**A**). **B** Plot of best-fitting 100 models for distinguishing future fallers from non-fallers based on AUC versus best-fitting models (Bayesian information criterion (BIC) best is zero on this scale) for ISAW metrics alone (color-coded by number of ISAW metrics) and fall history and age with ISAW metrics. The strongest models are circled in black with a list of the included ISAW metrics. Notice that sway (S) was the most commonly included ISAW domain

The model containing age and history of falls was strengthened by adding ISAW metrics. Fall history and age alone discriminated fallers from non-fallers with AUC 0.685 (0.611, 0.760) (Fig. 4A). Among the best-fitting 100 models, the median AUC among 3-metric models was 0.730, and the maximum AUC achieved was 0.750. The BIC-weighted model average of ISAW plus fall history and age had an AUC of 0.751 (0.680, 0.821). Age in this cohort alone, however, was not helpful in distinguishing fallers from non-fallers (AUC 0.551). When history of falls and age were included in each model, each additional ISAW metric, up to 3, gave a boost in AUC without substantial loss of model fit (Fig. 4B).

### Which ISAW domains help predict falls?

The models with the best combination of AUC and BIC included metrics from multiple different balance and gait domains of the ISAW, consistent with our hypothesis that several independent domains contribute valuable information about fall risk. The models with the best AUC each included a standing postural sway characteristic (e.g. larger sway velocity or higher sway frequency in fallers), whether fall history and age were included in the models or not. Likewise, models that included 2 ISAW metrics added slower turn velocity in fallers to increase AUC. When a third ISAW metric was added to the ISAW model, the best AUCs came from adding gait variability (e.g. increased gait cycle duration CoV in fallers) to sway and turning metrics. When a fourth or fifth ISAW metric was added to both types of models, it was most often the anticipatory postural adjustment prior to gait initiation (smaller amplitude in fallers).

A measure of the value of each ISAW metric within the seven mobility domains in predicting falls is given in Table 3. Specifically, this table gives the posterior probability that each ISAW metric is included in the “true” model, for the scenario in which only ISAW metrics are included in models, and the scenario in which fall history and age are also included. As we can see, when only ISAW metrics are used to predict future fallers, sway velocity, which is positively correlated with fall history (Spearman’s *r* = 0.18) and not an important predictor when fall history is known, becomes the most valuable metric. Turning velocity, which is negatively correlated with fall history (*r* =  − 0.08), also becomes much more valuable when fall history is not known. Stride time variability, which is uncorrelated with fall history (*r* = 0.01), also becomes more valuable in predicting falling. However, when fall history and age are included in the models, frequency of sway is the most important predictor of falling; it has the highest posterior probability of inclusion in the model and is uncorrelated with fall history, which suggests that it may capture an aspect of fall risk not explained by fall history.

**Table 3:** Posterior inclusion probabilities of each ISAW metric in the BIC-weighted model average, in which only ISAW metrics are predictors and when fall history and age are also included in models. Probabilities above 0.40 are shown in bold

Domain	Metric	ISAW metrics only	Fall history + age + ISAW metrics
Sway	Centroidal frequency	0.429	0.334
	Jerkiness	0.109	0.206
	Velocity	0.142	0.635
	Coronal RMS	0.076	0.102
APA*	Lateral peak	0.208	0.253
Gait/temporal	Stride time	0.112	0.087
	Double support time	0.081	0.083
Gait/spatial	Speed	0.154	0.202
	Stride length	0.095	0.130
Gait/variability	Stride time CoV	0.270	0.403
	Stride length CoV	0.074	0.075
	Lateral step deviation SD	0.194	0.205
Gait:	Coronal ROM	0.208	0.103
Upper body	Sagittal ROM	0.078	0.083
	Arm ROM	0.092	0.109
Turn	Duration	0.112	0.137
	Velocity	0.255	0.569

*APA* anticipatory postural adjustment, *RMS* root mean square, *CoV* coefficient of variation, *SD* standard deviation

### Do gait speed or dual-task cost improve fall prediction?

Although gait speed is the gait metric most often related to fall risk in the literature, it did not appear often in our top-performing fall prediction models. Therefore, we asked, specifically, how well gait speed alone, either as collected from stopwatch time of the 6-m walk (averaged across 2 trials) or from the ISAW 14-m walk, predicted future fallers. As expected, the stopwatch and ISAW gait speeds were highly correlated (*r* = 0.86; *p* < 0.001). However, neither stopwatch gait speed nor ISAW gait speed (stride velocity) predicted future fallers on their own (AUC = 0.589 and 0.595, respectively).

Adding a cognitive task while walking tended to reduce gait speed among participants, and the cost of the dual task on gait speed was very similar in future fallers and non-fallers. Mean dual task cost on gait speed was 20.53% and 19.99% among fallers and non-fallers, respectively (*p* < 0.001 in each case), and the dual task was associated with slowing of gait for all but 7 in each group.

In contrast to dual-task cost on gait speed, dual-task cost on turn velocity during the ISAW did improve prediction of future fallers from AUC 0.63 for ISAW metrics alone to AUC 0.745–0.764. This AUC for ISAW metrics during single-task walking plus dual-task cost on turning speed is slightly better than AUC for ISAW metrics combined with fall history and age (AUC 0.75 for BIC average, above).

Interestingly, approximately half the participants slowed down their turns while dual-tasking while the other half sped up their turns. Surprisingly, most participants who significantly slowed down their turns while performing the serial subtraction task were non-fallers, whereas most participants who significantly sped up their turns while dual-tasking were fallers. Non-fallers slowed turns by 13.33% while dual-tasking, whereas faller dual-task cost was only 3.14%. Turning velocity dual-task cost, alone, predicted falling quite well (AUC 0.629) compared to gait speed alone (AUC 0.504).

## Discussion

This is the first study to examine the contribution of objective gait as well as balance performance, across several different domains, to fall risk. We found that the Instrumented Stand and Walk (ISAW) test that quantified 17 characteristics across 3 balance domains (standing postural sway, APAs for step initiation, and turning) and 4 gait domains (spatial, temporal, variability, and upper body) predicted future fallers and significantly improved ability to predict who would fall in the next year above predictions from history of falls and age, alone.

The ISAW models that best predicted future fallers most often included measures of postural sway and adding turning velocity, gait variability, and anticipatory postural adjustments (APAs), as we hypothesized, based on the independence of these domains of mobility [6]. Unlike ISAW measures, gait speed, even when challenged with a dual (cognitive) task, was not a good predictor of who would fall, although dual-task cost on turning speed was a good predictor.

We decreased the risk of overfitting statistical models by first narrowing the set of gait and balance measures, and then averaging over thousands of models for fall risk that included every possible combination of ISAW metrics. Thus, rather than promoting a single combination of ISAW metrics to predict falling, we present results from an average of the models and present examples of top-performing models to examine which ISAW metrics were contributing.

History of falls in the past year was able to discriminate between prospective fallers and non-fallers reasonably well (AUC 0.695), consistent with previous studies in older adult participants [25]. However, ISAW metrics alone were able to predict falling slightly better than fall history (AUC 0.715 for model average with ISAW metrics alone). ISAW metrics also improved fall prediction when added to fall history and age (AUC 0.751 for the model average), suggesting that objective measures of balance and gait capture fall risk factors not apparent from fall history. Unlike fall history, ISAW metrics provide specific balance and gait targets for rehabilitation to prevent falls. Age, alone, did not provide good discrimination between fallers and non-fallers in this cohort (AUC 0.551). For this group of mostly white men with very good to excellent self-reported health, the age range was limited, but not trivial (78–96 years), and these older participants appeared particularly fit based on their PASE scores [23, 26, 27].

The impact of comorbidities on gait and balance may be captured by ISAW metrics. We did not include comorbidities in the modeling process since the focus of this paper is on the ability of ISAW metrics alone, and with age and fall history, to predict falling in a cohort with little comorbidity. Also, the available data on comorbidities has limitations due to self-report, which could make results misleading. Future studies should investigate whether adding comorbidities to the fall prediction model would improve performance.

### Gait speed and dual-task gait

Surprisingly, gait speed was not represented in our fall prediction models very often, although it is the most frequently cited and most easily measured gait feature [9, 28]. Gait speed alone did not distinguish between fallers and non-fallers (AUC only 0.595). However, this high-functioning cohort of older men had a mean gait speed of 1.19 m/s (interquartile range (1.04, 1.33), so they would not be considered high risk for a fall based on gait speed, alone [29].

In addition, dual-task cost on gait speed while serially subtracting was not helpful for fall prediction. Dual-task cost may not have been helpful because of high cognitive function in this cohort who had no trouble walking while subtracting by threes. However, dual-task cost on turning speed did predict future fallers, but not as expected. Fallers tended to increase their turning speed while dual-tasking, unlike non-fallers, who slowed down. Speeding up turns so the mental task that can be accomplished may be a maladaptive strategy that leads to more falls. In fact, dual-task cost on turning velocity improved prediction of future fallers from AUC 0.63 for ISAW metrics alone to AUC 0.764. This AUC for ISAW metrics during single-task walking plus dual-task cost on turning speed was even better than the AUC for ISAW metrics combined with fall history (AUC 0.75) suggesting that fall history reflects real-world challenges of dividing attention while performing difficult mobility tasks, such as turning.

### Balance and gait domains

The ISAW was specifically designed to include several different domains of balance and gait in a short (< 1 min), clinical protocol appropriate for clinical trials or clinical practice. Laboratory and home studies of balance and gait have suggested that postural sway characteristics (area, velocity, and frequency), gait characteristics (stride time variability, double support time, trunk range, and gait speed), and turning characteristics (peak velocity, number of steps, variability) differ in older adult fallers versus non-fallers [30]. Studies have shown that balance control for sway in quiet stance relies upon different neural control systems than control of gait [6] or from control of APAs [31]. In fact, straight-ahead gait, itself, consists of several different domains, since factor analysis consistently shows separate domains for spatial (pace/speed), timing, variability, and upper body control [8]. Our results are consistent with our hypothesis that several different domains of the ISAW test can help predict future fallers.

Most studies examining the relationship between mobility metrics from body-worn sensors and fall risk have focused on gait [30, 32, 33]. However, in our study, postural sway metrics in standing were more frequent in our fall-prediction models than gait metrics. Surprisingly, sway metrics most likely to be included in the models were sway velocity or frequency, not sway area [34, 35]. Postural sway represents the output of a complex multisensory (vision, vestibular, and somatosensory) multi-motor (corticospinal, reticulospinal, vestibulospinal) control system for control of postural equilibrium. Increases in postural sway velocity or frequency may reflect more frequent balance corrections needed to maintain equilibrium [36]. The most common reason for increased postural sway frequency or velocity in the older adults is proprioceptive neuropathy, followed by vestibular and/or visual loss, and muscle weakness, but we cannot distinguish which set of age-related impairments was present in our relatively healthy cohort [37]. Note that postural sway was measured with eyes open while standing on a firm surface with feet a standard distance apart, a simple condition that all of our participants could accomplish.

After postural sway, turning characteristics were most frequently presented in the models. This result is consistent with our previous study showing that turning measured during a week of daily life could separate older adult fallers from non-fallers [38, 39]. Turning is a complex dynamic balance task that requires reorientation of the oculomotor system, followed by a top-down axial rotation. The body center of mass passes close to its limits of foot stability during turns making it a high-risk, functional activity in people with compromised balance control [40]. In fact, many falls and fractures in older adults have been associated with turning [38, 39]. However, fallers (but not non-fallers) in our study increased their turning velocity when attention was diverted with a cognitive dual-task, suggesting poor compensation for their dynamic balance deficits, leading to increased fall risk.

When 3 or more ISAW metrics were added to the models, the lateral peak APA became helpful. Lateral weight-shift, necessary to unweight the stepping leg, is known to be critical for developing fast, long initial steps [41]. The size of the APA is normally tuned to the anticipated gait velocity, as well as to initial stance width, with larger APAs needed for larger stance widths. The ISAW test controlled standing stance width across all participants using a foot template [18, 41]. We found that larger APAs were more commonly associated with future fallers but know of no other studies relating APAs to falls. Excessively large APAs, however, can be seen in patients with Parkinson’s with freezing and people with neuropathy, common neurological disorders in older adults, and associated with frequent falls [42, 43]. The most common gait metric added to our fall prediction models was variability (COV) of stride time. Indeed, stride time variability has previously been found to be associated with past and future fallers, as it may represent compensatory stepping for postural corrections of imbalance while walking [32, 44].

### Significance of the ISAW test

Despite normal gait speed and normal dual-task cost, our mostly healthy, independent cohort of older men in their 80 s demonstrated many abnormalities of standing balance, gait initiation, turning (and dual-task turning), and gait characteristics that can contribute to their fall risk. Although gait speed alone did not distinguish between fallers versus non-fallers (AUC = 0.589) in this cohort, ISAW measures alone did much better (AUC = 0.715). Most of the objective measures from the ISAW cannot be readily observed with clinical tests of balance or gait. ISAW testing could be useful for those without a history of falling, those who cannot reliably recall past falls, and those who should be referred to fall risk reduction physical therapy so the therapists can focus their exercises on the specific domain of balance or gait affected [45]. Unlike clinical tests of balance and gait that rate ability to accomplish functional tasks, the ISAW provides objective measures of specific balance and gait domain impairments that document how and why each individual’s task performance is impaired [46–48].

### Limitations

This study has two main limitations. First, the individual models are not yet validated in a separate cohort so performance of the models considered is optimistic. However, our approach yielded a weighted average across many models, including those with good and poor performance, and this averaging across models tempers overfitting. Second, our cohort consisted of white, high-functioning, mostly community-dwelling men at one site. The relative homogeneity of the cohort in terms of gender, age, race/ethnicity, geographic area, and good-to-excellent self-reported health limited the external generalizability of the results. Nevertheless, the ISAW helped to predict the 40% who were fallers, despite their good health, normal gait speed, and normal dual-task cost on gait speed. In view of these limitations, we believe that external validation with a larger, more heterogeneous sample is needed to determine the best ISAW model for fall prediction, and perhaps even to discriminate frequent fallers from single fallers. Although the specific, best ISAW metrics for fall prediction may vary per cohort, we predict multiple gait and balance domains would be represented.

## Conclusions

The ISAW test, based on wearable inertial sensor measurements of three balance and four gait domains, predicted falls as well as fall history and improved prediction of future fallers when added to fall history. The best models most often included measures of postural sway, gait variability, turning speed, and gait initiation, consistent with our hypothesis that older people may fall due to a variety of balance and gait impairments.
